# Inhibitory Effects of Cu_2_O/SiO_2_ on the Growth of *Microcystis aeruginosa* and Its Mechanism

**DOI:** 10.3390/nano9121669

**Published:** 2019-11-22

**Authors:** Gongduan Fan, Minchen Bao, Bo Wang, Shimin Wu, Lingxi Luo, Binhui Li, Jiuhong Lin

**Affiliations:** 1 College of Civil Engineering, Fuzhou University, Fujian 350116, China; N170527002@fzu.edu.cn (M.B.); n190527070@fzu.edu.cn (J.L.); 2 IER Environmental Protection Engineering Technology Co., Ltd., Shenzhen 518071, China; wushimin@gmail.com (S.W.); luolingxi1985@outlook.com (L.L.); libinhui2727@outlook.com (B.L.); 3 School of Urban Planning and Design, Shenzhen Graduate School, Peking University, Shenzhen 518055, China

**Keywords:** harmful cyanobacteria, Cu_2_O/SiO_2_, hydroxyl radical, antioxidant enzyme

## Abstract

In this study, a novel nanomaterial Cu_2_O/SiO_2_ was synthesized based on nano-SiO_2_, and the inhibitory effects of different concentrations of Cu_2_O/SiO_2_ on the growth of *Microcystis aeruginosa* (*M. aeruginosa*) were studied. At the same time, the mechanism of Cu_2_O/SiO_2_ inhibiting the growth of *M. aeruginosa* was discussed from the aspects of Cu^2+^ release, chlorophyll *a* destruction, oxidative damage, total protein, and the phycobiliprotein of algae cells. The results showed that low doses of Cu_2_O/SiO_2_ could promote the growth of *M. aeruginosa*. When the concentration of Cu_2_O/SiO_2_ reached 10 mg/L, it exhibited the best inhibitory effect on *M. aeruginosa*, and the relative inhibition rate reached 294% at 120 h. In terms of the algae inhibition mechanism, Cu_2_O/SiO_2_ will release Cu^2+^ in the solution and induce metal toxicity to algae cells. At the same time, *M. aeruginosa* might suffer oxidative damage by the free radicals, such as hydroxyl radicals released from Cu_2_O/SiO_2_, affecting the physiological characteristics of algae cells. Moreover, after the addition of Cu_2_O/SiO_2_, a decrease in the content of chlorophyll *a*, total soluble protein, and phycobiliprotein was found, which eventually led to the death of *M. aeruginosa*. Therefore, Cu_2_O/SiO_2_ can be used as an algaecide inhibitor for controlling harmful cyanobacteria blooms.

## 1. Introduction

Excess nutrients (e.g., nitrogen and phosphorus) in the environment lead to the outbreak of harmful algal blooms (HABs) and the pollution of water sources [1], which caused wide concern [2]. HABs have a huge impact on human lives, production, and health. The cyanobacteria that spreads over the surface of the water will increase the turbidity of the lake and release some “unpleasant” taste to reduce the ornamental value of the water. The excessive reproduction and growth of cyanobacteria will consume dissolved oxygen, reduce the transparency of water, and change the pH, which has already threatened the human’s production and lives and the survival of aquatic animals and plants. What is worse, the death of cyanobacteria will release a variety of toxins (e.g., hepatotoxin and neurotoxin) that can cause serious long-term damage to human health [3].

Treatment schemes for controlling HABs have focused on the physical, chemical, and biological methods [4,5,6]. Most of those methods show promise, but hard to achieve safe and efficient applications in natural water bodies. In recent years, nanomaterials were widely used in algal growth inhibition, on account of the advantages of non-toxicity, high chemical stability, and strong adsorption capacity. Wang et al. [7] observed that 100 mg/L TiO_2_ will significantly inhibit the growth of *Chlamydomonas*. Zhao et al. [8] found that the growth inhibition rate of *Chlorella pyrenoidosa* exposed to 200 mg/L GO was more than 80%. Gu et al. [9] reported that the chlorophyll *a* removal rate of *M. aeruginosa* reached 80.6% in 2.5 h using 0.25 g/L Zn-Fe LDHs under visible light in photoreaction instrument equipped with a 300 W xenon lamp. Nevertheless, it must eliminate these defects, such as high cost, large dose, and the dependence on high-intensity irradiation to promote the nanomaterials applied as algicides in natural waters.

Cu-based nanomaterials can continuously inhibit the growth of cyanobacteria due to the characteristics that algae were sensitive to Cu^2+^ [10]. Cu_2_O and modified-Cu_2_O exhibit excellent photodegradability under low-intensity visible light [11,12], which makes it an ideal Cu-based nanomaterial for controlling HABs. Cu_2_O is a common p-type semiconductor with a band gap of 2.0 to 2.2 eV. Cu_2_O has attracted wide attention in the fields of catalysis, lithium batteries and sensors due to its good visible light response [13,14]. However, the e^−^/h^+^ pairs on Cu_2_O cannot efficiently separate, which will lead to the fast recombination of the e^−^/h^+^ pairs, thus resulting in a decrease of the photocatalytic activity [15]. As a porous hydrophilic material, nano-SiO_2_ has an -OH group on its surface and is often used as a matrix for nanomaterials [16]. The modification of Cu_2_O with SiO_2_ will increase the adsorption of organic substances on the surface acid sites and prevent the recombination of electron-hole pairs, which can improve the photocatalytic performance of Cu_2_O/SiO_2_. Besides, SiO_2_ is commercially available and inexpensive, which makes the manufacturing cost of Cu_2_O/SiO_2_ greatly low.

Revealing the mechanism of growth inhibition of cyanobacterial cells by Cu_2_O/SiO_2_ will be helpful for its application in controlling HABs. Metal-containing nanomaterials will release metal ions into the solution, which can affect the growth of algae. Douglas et al. [17] found that the photosynthetic activity of *Dinoflagellates* decreased with increasing Cu^2+^ concentration. Franklin et al. [18] believed that Zn^2+^ released by ZnO will inhibit the growth of *Selenastrum bibraianum*. Sunandan et al. [19] found that Al^3+^ released by nano-Al_2_O_3_ reduced the cell viability of algae. Fan et al. [20] found that Cu^2+^ released by Cu-MOF-74 plays an important role on the growth inhibition of *M. aeruginosa*. Besides, nanomaterials could produce reactive oxygen species (ROSs) when in contact with the microorganisms. Common ROSs include H_2_O_2_, ·OH, and ·O_2_^−^ [21,22]. When ROS were produced inside the cell, the antioxidant enzyme system, such as superoxide dismutase (SOD) and catalase (CAT), was activated to resist ROS and protect cells from oxidative damage. However, when the ROS content is too high for the cells to repair themselves, it will cause oxidative damage to the algae cells [23,24,25]. ·OH can react with terephthalic acid and then form 2,5-dihydroxyterephthalic acid, which can be excited by incident light and exhibit luminescence at 315 nm. The content of ·OH in the solution can be judged based on the fluorescence intensity [26,27]. Isopropyl alcohol (IPA) can inhibit the release of ·OH from nanomaterials, so it can be used as a quencher for ·OH [28]. Wang et al. [29] found that the DHA activity of *M. aeruginosa* was significantly inhibited from 654.06 U/L to 177.94 U/L after contacting F-Ce-TiO_2_. Oukarroum et al. [30] found that the amount of ROS that was produced by *Chlorella* exposed to 10 mg/L nano-NiO for 96 h was 37 times higher than that of the control group. At the same time, the accumulation of ROS in algal cells led to lipid peroxidation, which caused the cell membrane to lose its integrity and selectively permeability [31]. The structure of the algae cells was destroyed, which caused a large amount of leakage of intracellular organic matter (IOM) and electrolytes, eventually leading to cell death.

The effect of nanomaterials on the photosynthetic system of algae cells includes two aspects: first, the effect on the light-harvesting ability of algae cells; second, the effect on chlorophyll. Algae are a kind of photoautotrophic organisms that undergo photosynthesis through photosynthetic pigments. Chlorophyll and phycobilin (PB) are the most important photosynthetic pigments in algae cells and they play an important role in photosynthesis. The PB is the main photosynthetic pigment in cyanobacteria and it usually combines with proteins to form phycobiliproteins (PBPs). The PBPs of *M. aeruginosa* can be divided into phycocyanin (PC), phycoerythrin (PE), and allophycocyanin (APC) [32]. Gu et al. [33] found that the removal rate of chlorophyll *a* by 0.4 g/L Cu_2_O-montmorillonite was as high as 90.2%, when Cu_2_O-montmorillonite was used to inhibit *M. aeruginosa*. Wang et al. [34] found that H_2_O_2_ inside the cells caused phycocyanin to detach from the thylakoid membrane.

In this study, a novel nanomaterial Cu_2_O/SiO_2_ was synthesized by the solution-phase method and characterized by X-ray diffraction (XRD), scanning electron microscope (SEM), and ultraviolet–visible spectroscopy (UV-Vis). Subsequently, the effects of Cu_2_O/SiO_2_ on photosynthetic pigments, total soluble protein, and phycobiliprotein of algae cells were studied. Finally, to reveal the mechanism of growth inhibition of *M. aeruginosa*, the interaction between Cu_2_O/SiO_2_ and algae cells were analyzed from the aspects of metal ion release, oxidative damage, and photosynthetic system damage. Overall, this study provides a theoretical basis for the application of Cu_2_O/SiO_2_ on control harmful cyanobacterial blooms.

## 2. Materials and Methods

### 2.1. Synthesis and Characterization of Cu_2_O/SiO_2_

Cu_2_O/SiO_2_ was prepared according to the following steps: 5 mL of CuCl_2_(0.5 mol/L) and 1.9 g of nano-SiO_2_ were first added to a mixture of 85 mL of deionized water and 20 mL of absolute ethanol. Afterwards, the mixture was transferred to a water bath at 40 °C. Later, 9 mL of NaOH (1 mol/L), 30 mL of absolute ethanol, and 9.8 mL of hydroxylamine hydrochloride were added and stirred well for 3 min. The mixture was centrifuged, washed, and dispersed in 50% ethanol and finally dried at 45 °C.

The synthesized Cu_2_O/SiO_2_ was characterized by scanning electron microscope (FEI, Helios G4 CX, Waltham, MA, USA), X-ray diffraction measurement (Rigaku, Ultima IV, Tokyo, Japan), and UV-Vis spectroscopy (PerkinElmer, LAMBDA 800 PE, Waltham, MA, USA).

### 2.2. Algae Growth Inhibition Experiment

#### 2.2.1. Algae Cultures

Cyanobacterial species of *M. aeruginosa* (FACHB 905) were obtained from the Freshwater Algae Culture Collection of the Institute of Hydrobiology (FACHB) located in China. The strain was grown in BG-11 medium at 30 °C with illumination at 2000 Lux under 14L/10D cycle.

#### 2.2.2. Cu_2_O/SiO_2_ Inhibit Algae Growth Experiment

The Cu_2_O/SiO_2_ stock solution (1000 mg/L) was obtained by adding 0.1 g of Cu_2_O/SiO_2_ into 100 mL deionized water. Subsequently, add 0, 0.01, 0.1, 0.5, 1, 2, and 5 mL of Cu_2_O/SiO_2_ stock solution to 100 mL of *M. aeruginosa* to achieve Cu_2_O/SiO_2_ concentrations of 0, 0.1, 1, 5, 10, 20, and 50 mg/L, respectively. Subsequently, algal cell density (OD_680_) was determined by ultraviolet-visible spectrophotometer (Persee, New Century T6, Beijing, China) at 24-h intervals. The growth inhibition rate was calculated, as follows:

(1) $$\mu_{i-j}=\frac{\ln X_{j}-\ln X_{i}}{t_{j}-t_{i}}$$

(2) $$I_{r}=\frac{\mu_{C}-\mu_{T}}{\mu_{C}}\times 100\%$$

where, X_j_ is the OD_680_ value of the sample at j (h); X_i_ is the OD_680_ value of the sample at i (h); μ_i-j_ (%) is the specific growth rate of the sample; μ_C_ (%) is the average of the specific growth rates of the control group; μ_T_ (%) is the average of the specific growth rates of the experimental groups; and, I_r_ (%) is the inhibition rate that is based on the specific growth rate.

### 2.3. Mechanism Experiment of Cu_2_O/SiO_2_ Inhibiting Algae Growth

#### 2.3.1. The Effect of Cu^2+^

Firstly, 1 mL of Cu_2_O/SiO_2_ stock solution (1000 mg/L) was added to 100 mL of deionized water to obtain a 10 mg/L Cu_2_O/SiO_2_ solution. Subsequently, the samples were filtered (0.22 μm membrane filter) and the Cu^2+^ concentration was then measured while using an inductively coupled plasma spectrometer (PerkinElmer, Optima 8000, USA) to determine the concentration of Cu^2+^ released from 10 mg/L Cu_2_O/SiO_2_. Finally, CuSO_4_·5H_2_O was selected as another source of Cu^2+^ to compare the effect of Cu^2+^ and Cu_2_O/SiO_2_ on the growth of *M. aeruginosa*. The concentration of CuSO_4_·5H_2_O in this experiment was the same as the concentration of Cu^2+^ released by 10 mg/L Cu_2_O/SiO_2_.

#### 2.3.2. The Effect on Chlorophyll *a*

Cu_2_O/SiO_2_ stock solution was added to the *M. aeruginosa* and cultured under the same conditions in Section 2.2.2. The samples were taken every 24 h and the chlorophyll *a* content of the samples was spectrometrically determined according to the Chinese Environmental Protection Agency standard method [35]. The relative content of chlorophyll *a* was calculated by Equation (3).

Relative content of chlorophyll *a* (%) = (C/C_0_) × 100 (3)

#### 2.3.3. The Release of ·OH

Dissolve 2.0 mM terephthalic acid and excess NaOH in 400 mL of deionized water and stirred to fully dissolve. Subsequently, the pH of the solution was adjusted to approximately 7.0 with HCl. The above solution was evenly divided into two parts. Subsequently, Cu_2_O/SiO_2_ was added to the two groups of solutions, and 1 mM IPA was added to the control solution. After 2 h of the experiment, an aliquot of 5 mL of the sample was centrifuged at 3000× *g* for 5 min. Finally, the fluorescence intensity of the supernatant was measured with a fluorescence spectrometer (Edinburgh, FS5, Edinburgh, UK). Excitation (Ex): 250–450 nm in 5 nm steps, Emission (Em): 350–450 nm in 5 nm steps.

#### 2.3.4. Active Species Trapping Experiments

For active species trapping experiments, isopropyl alcohol (IPA), benzoquinone (BQ), and sodium oxalate (SO), were utilized to trap ·OH, superoxide radicals ·O_2_^−^, and h^+^, respectively. The concentration of the scavengers was 0.01 mol/L. The whole process was designed similarly to Section 2.2.2.

#### 2.3.5. The Effect on Antioxidant Enzyme

Firstly, 5 mL of the sample was centrifuged at 3000× *g* for 10 min. and the algal cell pellets were washed three times with PBS buffer solution. Then, the pellets were disrupted ultrasonically at 4 °C for 10 min. with 3 s pulses at 200 W by a cell sonicator (SCIENTZ JY92-II, Ningbo, China). Subsequently, in order to obtain the cell-free crude extract, algal cell debris was removed by centrifugation (3000× *g* for 10 min. at 4 °C). Finally, antioxidative enzymes (Superoxide dismutase, SOD; Catalase, CAT) were spectrophotometrically measured with commercial-available kits (Nanjing Jiancheng Bioengineering Institute, Nanjing, China).

#### 2.3.6. Extracellular Organic Matter (EOM) & Intracellular Organic Matter (IOM)

The extracellular organic matter (EOM) and intracellular organic matter (IOM) of *M. aeruginosa* were extracted by the method of Tian and Qu et al. [36,37]. Fluorescence measurement of the EOM & IOM extract was conducted by using a fluorescence spectrometer; Ex was scanned from 200 to 450 nm, with a step of 5.0 nm, and Em from 200 to 550 nm with a step of 5.0 nm.

#### 2.3.7. The Effect on Total Protein

The extraction method of the total protein (TP) from *M. aeruginosa* is referred to as the method of Tao et al. [38]. The coomassie blue staining method determined the concentration of TP while using an assay kit (#A405-2-2, Nanjing Jiancheng Bioengineering Institute, Nanjing, China).

#### 2.3.8. The Effect on Phycobiliprotein

The extraction method of phycobiliprotein from *M. aeruginosa* is referred to as the method of Padgett et al. [39]. Firstly, 8 mL of the sample was centrifuged at 3000× *g* and 4 °C for 15 min. and the supernatant was discarded. Subsequently, the algal cells were washed and resuspended in 4 mL of PBS followed by being frozen and rapidly thawed three times. After centrifugation, the absorbance of the supernatant was finally measured at 565 nm, 620 nm, and 650 nm with a spectrophotometer.

The contents of PC, PE, APC, and PB were calculated, as follows:

(4) $$PC\left(mg/L\right)=\frac{OD_{620}-0.7\times OD_{650}}{7.38}$$

(5) $$APC\left(mg/L\right)=\frac{OD_{650}-0.19\times OD_{620}}{5.65}$$

(6) $$PE\left(mg/L\right)=\frac{OD_{565}-2.8\times PC-1.34\times APC}{1.27}$$

PB = PE + PC + APC(7)

## 3. Results and Discussion

### 3.1. Characterization of Cu_2_O/SiO_2_

#### 3.1.1. X-ray Diffraction (XRD) & Scanning Electron Microscope (SEM) 

**Figure 1:**
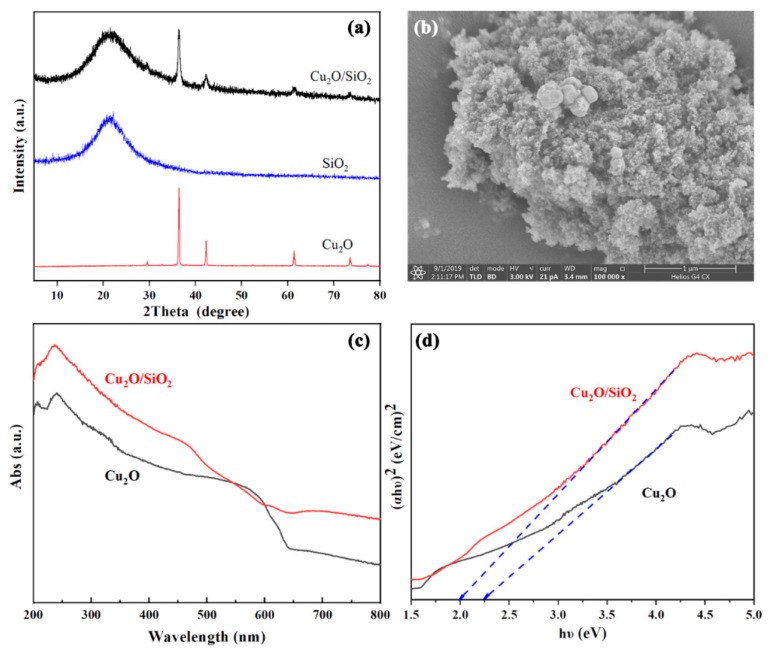
(**a**) X-ray diffraction (XRD) pattern of Cu_2_O, SiO_2_ and Cu_2_O/SiO_2_; (**b**) scanning electron microscope (SEM) image of Cu_2_O/SiO_2_; (**c**) UV-Vis patterns of Cu_2_O and Cu_2_O/SiO_2_; and, (**d**) Tauc plot for pure Cu_2_O and Cu_2_O/SiO_2_.

Figure 1a shows the XRD patterns of Cu_2_O, SiO_2_ and Cu_2_O/SiO_2_. As can be seen from the Figure 1, there are five distinct diffraction peaks of pure Cu_2_O (2*θ* = 29.5°, 36.3°, 42.3°, 61.3°, and 73.5°). The Cu_2_O/SiO_2_ synthesized by the solution phase method contains all of the characteristic peaks of Cu_2_O, which indicates the successful synthesis of Cu_2_O/SiO_2_. Figure 1b shows the SEM image of Cu_2_O/SiO_2_. A large number of porous particles can be observed, because nano-SiO_2_ covers the surface of Cu_2_O. This is consistent with the morphology of the Cu_2_O-SiO_2_ nanoparticles observed by Xia et al. [40].

#### 3.1.2. Ultraviolet-Visible Spectroscopy (UV-Vis)

Figure 1c provides the UV-Vis absorption spectra of the Cu_2_O and Cu_2_O/SiO_2_. After coupling with SiO_2_, the Cu_2_O/SiO_2_ exhibits strong absorption both in UV and visible light with a wavelength shorter than 480 nm, indicating the dramatically increased optical absorption of Cu_2_O. The bandgap energy of pure Cu_2_O and Cu_2_O/SiO_2_ were calculated through a Tauc plot of (*αhυ*)^2^ vs. *hυ*, as shown in Figure 1d. In accordance with formula (8), the band gap energy of Cu_2_O and Cu_2_O/SiO_2_ were calculated to be 2.24 and 2.01 eV, respectively. The narrowed bandgap indicated the possibility of Cu_2_O/SiO_2_ to enhance photocatalytic activity.

*αhυ* = K(*hυ*-Eg)^1/2^ (8)

where, *α* is the absorption coefficient, K represents a constant, *hυ* stands for the discrete photo energy, and Eg is the band gap energy.

### 3.2. Growth Inhibition of Cu_2_O/SiO_2_ on Algal Cells

**Figure 2:**
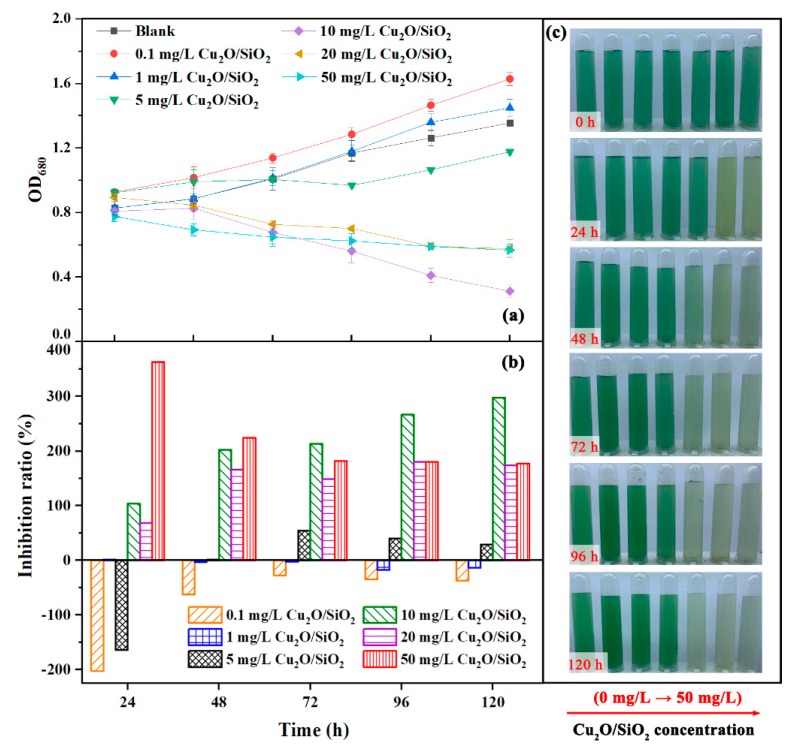
The growth of *M. aeruginosa* after treatment with different concentrations of Cu_2_O/SiO_2_. (**a**) OD_680_ value; (**b**) Inhibition rate; and, (**c**) The color change of algae during the experiment.

Figure 2 shows the growth situation of *M. aeruginosa* after treatment with different concentrations of Cu_2_O/SiO_2_ during the experiment for the first time. As shown in Figure 2c, algae exposed to 10, 20, and 50 mg/L Cu_2_O/SiO_2_ had varying degrees of fading during the experiment. It can be seen from Figure 2a that the OD_680_ value of *M. aeruginosa* in the control group gradually increased during the experiment, indicating that the algae cells were in good condition. Low concentrations of Cu_2_O/SiO_2_ (0.1, 1, and 5 mg/L) may promote the growth of *M. aeruginosa*, presumably because low doses of Cu^2+^ will promote the growth of *M. aeruginosa*. Duong et al. [17] found that 0.001 mg/L nano-Ag could not inhibit the growth of *M. aeruginosa*. The OD_680_ value of *M. aeruginosa* exposed to 10, 20, and 50 mg/L Cu_2_O/SiO_2_ showed a downward trend, and 10 mg/L Cu_2_O/SiO_2_ had the strongest inhibitory effect on the growth of *M. aeruginosa*. The OD_680_ value of *M. aeruginosa* exposed to 20 and 50 mg/L Cu_2_O/SiO_2_ was higher than 10 mg/L Cu_2_O/SiO_2_ in the later stage of the experiment, which was probably due to the absorbance of high concentration nanomaterials. As shown in Figure 2b, the inhibition rate of Cu_2_O/SiO_2_ to *M. aeruginosa* gradually increased during the experiment, which could reach 297% at 120 h, indicating that Cu_2_O/SiO_2_ could inhibit the growth of the algae to some extent.

### 3.3. Mechanism of Cu_2_O/SiO_2_ Inhibiting Algae Growth

#### 3.3.1. Metal Ions

**Figure 3:**
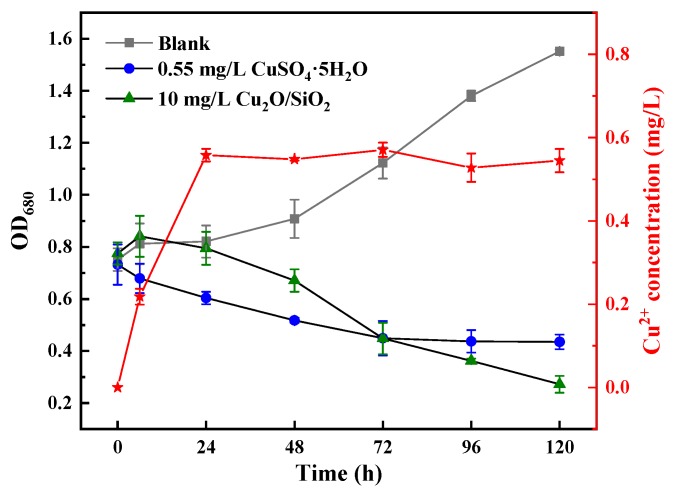
The concentration of Cu^2+^ released by Cu_2_O/SiO_2_ in solution and its effect on the growth of *M. aeruginosa*.

The red line in Figure 3 shows the Cu^2+^ concentration released by 10 mg/L Cu_2_O/SiO_2_ in the solution. The amount of Cu^2+^ released by Cu_2_O/SiO_2_ increased at the beginning of the experiment and reached equilibrium at 24 h (about 0.55 mg/L). The OD_680_ values of *M. aeruginosa* that were exposed to Cu_2_O/SiO_2_ and CuSO_4_·5H_2_O(Cu^2+^) are shown in the black line portion of Figure 3. The control group grew normally during the experiment, and the growth of *M. aeruginosa* exposed to 0.55 mg/L CuSO_4_·5H_2_O was immediately inhibited. The OD_680_ of *M. aeruginosa* exposed to 10 mg/L Cu_2_O/SiO_2_ initially increased and then rapidly decreased, eventually reaching 0.272. The inhibitory effect of 10 mg/L Cu_2_O/SiO_2_ on *M. aeruginosa* was higher than that of 0.55 mg/L Cu_2_O/SiO_2_, so the release of metal ions is considered to be a part of the mechanism of metal-containing nanomaterials inhibiting algae growth.

#### 3.3.2. Chlorophyll *a*

**Figure 4:**
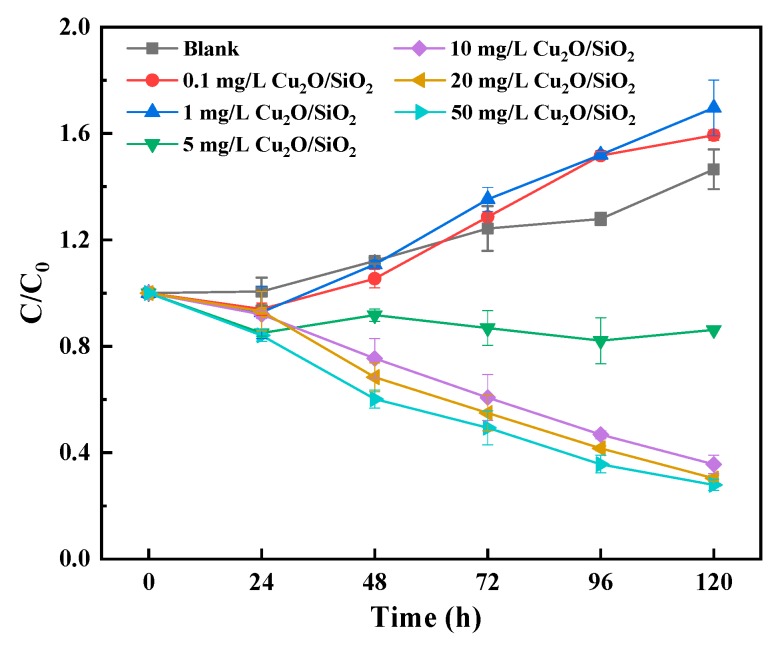
Effects of different concentrations of Cu_2_O/SiO_2_ on the content of chlorophyll *a* in *M. aeruginosa*.

Figure 4 shows the trend of the relative content of chlorophyll *a* during the experiment. The chlorophyll *a* content can also reflect the growth of *M. aeruginosa* from another side. By observing Figure 2a and Figure 4, it can be found that the OD_680_ value and the relative content chlorophyll *a* of *M. aeruginosa* have the same trend of variability during the experiment. The content of chlorophyll *a* of the *M. aeruginosa* in the control group grew continuously during the experiment, indicating that the algal cells in the control group grew well. The content of chlorophyll *a* in the algae cells that were exposed to 0.1 and 1 mg/L Cu_2_O/SiO_2_ increased with time and even exceeded the chlorophyll *a* content of the control group at 72–120 h, which indicates that low doses of Cu_2_O/SiO_2_ had no effect on the chlorophyll *a* of *M. aeruginosa*. The content of chlorophyll *a* in algae cells that were exposed to 10, 20, and 50 mg/L Cu_2_O/SiO_2_ decreased significantly and it was only about 25% of the initial content at 120 h. Similar results that were obtained by Hu et al. [41] showed that high doses of TiO_2_ would inhibit the production of chlorophyll *a* in *Chrysophyta*. Based on the results above, it can be found that high doses of Cu_2_O/SiO_2_ (above 10 mg/L) would cause a decrease in the relative content chlorophyll *a* of *M. aeruginosa* and affect the growth of algae cells.

#### 3.3.3. ·OH Assays

**Figure 5:**
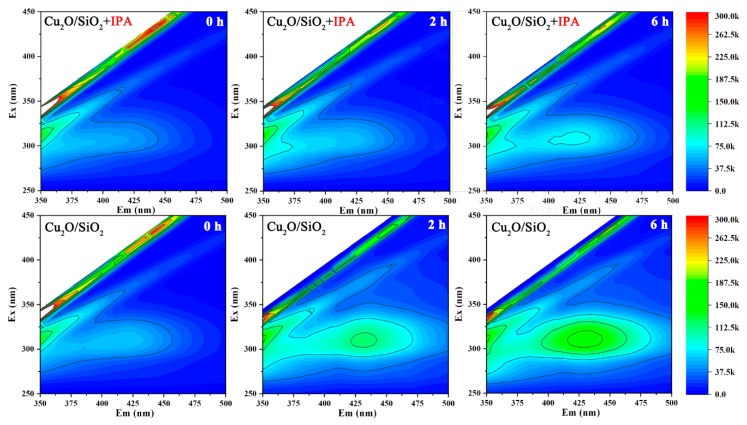
Excitation-emission matrix (EEM) diagram of ·OH concentration change with time.

Figure 5 shows the fluorescence spectrum of the Cu_2_O/SiO_2_ suspension. It can be found from Figure 5 that the experimental group showed an obvious fluorescence peak at 315 nm at 2 h and 6 h. The intensity of this fluorescent peak became stronger over time, indicating that continuous illumination will promote the production of ·OH by Cu_2_O/SiO_2_. The intensity of the fluorescent peak of the control group was significantly lower than that of the experimental group, because ·OH was continuously quenched by isopropanol. From this experiment, it can be found that, when being added in the solution, Cu_2_O/SiO_2_ would produce a small amount of ·OH immediately, and the amount of ·OH would increase with the increase of illumination time.

#### 3.3.4. Active Species Trapping Experiments

**Figure 6:**
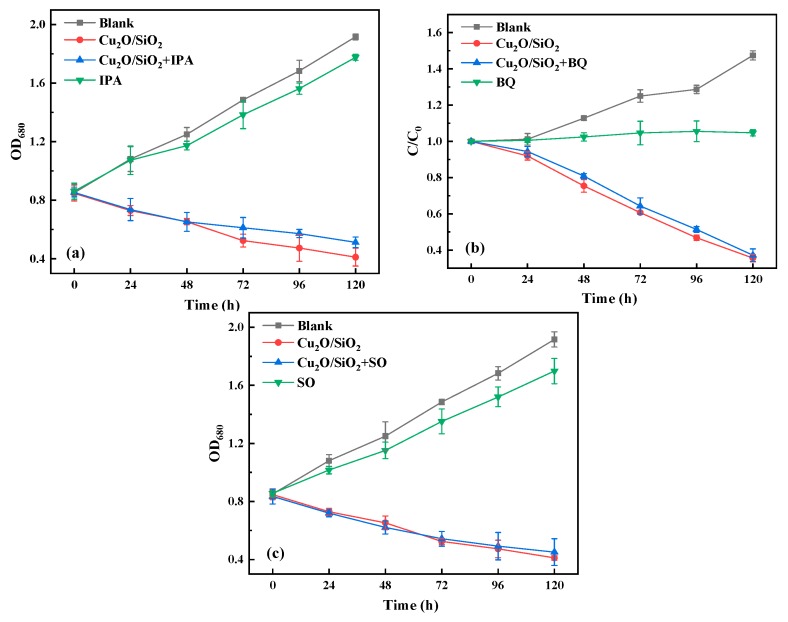
Active species trapping experiments. (**a**) ·OH; (**b**) ·O_2_^−^; and, (**c**) h^+^.

Figure 6 shows the trends of the three kinds of ROS during the active species trapping experiments. As shown in Figure 6, IPA and SO have a slight effect on the growth of algae cells, while BQ could inhibit the growth of algae cells. The relative inhibition rate of Cu_2_O/SiO_2_ to *M. aeruginosa* decreased from 189% to 162% after the addition of IPA. However, the inhibitory effect of Cu_2_O/SiO_2_ on the growth of *M. aeruginosa* after adding BQ and SO was not significantly different from that of pure Cu_2_O/SiO_2_. Therefore, it can be inferred that ·OH is the main active substance in the experiment based on the experimental results.

#### 3.3.5. Antioxidant Enzyme

**Figure 7:**
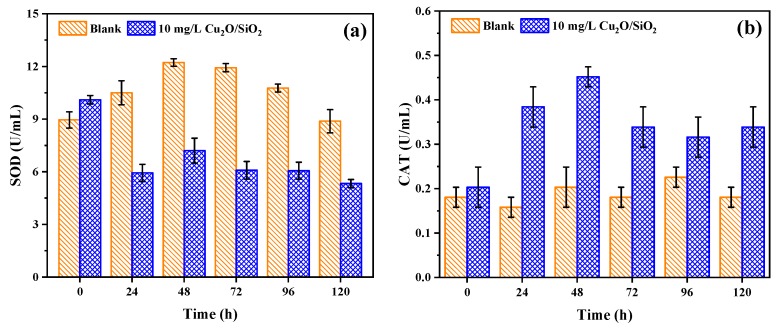
Changes of (**a**) superoxide dismutase (SOD) and (**b**) catalase (CAT) activities during algae removal by Cu_2_O/SiO_2_.

Figure 7 shows the effect of Cu_2_O/SiO_2_ on the activities of antioxidant enzymes of *M. aeruginosa*. The SOD activity of the control group algae floated within a certain range, while the SOD activity of the experimental group sharply decreased at the beginning of the experiment and remained relatively stable thereafter. It indicates that, when exposed to Cu_2_O/SiO_2_, the SOD of *M. aeruginosa* began to scavenge free radicals to inhibit oxidative damage. This is consistent with the study by Hazani et al. [42]. They found a significant decrease in the SOD activity of *Chlorella* and *Dunaliella salina* that were exposed to 100 and 200 mg/L of nano-Ag. The change in CAT activity of algal cells during the experiment is shown in Figure 7b. It can be found that the CAT activity of *M. aeruginosa* rapidly rose and then remained at a higher level as the experiment progressed. It is speculated that, in the early stage of the experiment, Cu_2_O/SiO_2_ will cause an increase in H_2_O_2_ content within algae cells, thus promoting CAT activity to protect against oxidative stress.

#### 3.3.6. EOM & IOM

**Figure 8:**
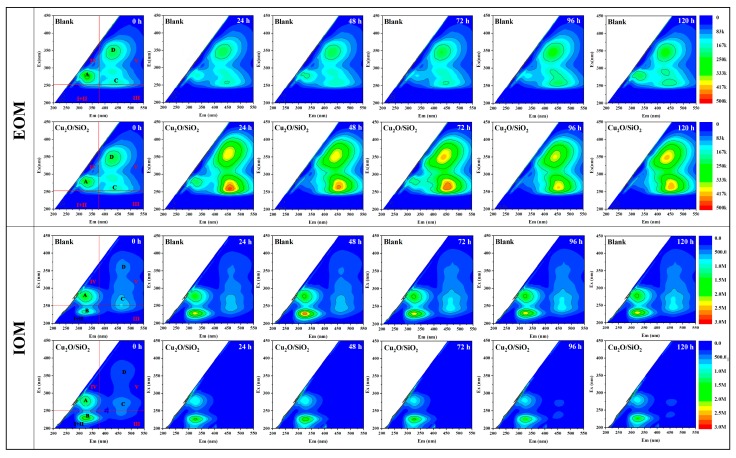
The fluorescence spectrum of extracellular organic matter (EOM) & intracellular organic matter (IOM) of algae cells.

Figure 8 presents the fluorescence spectra of EOM and IOM of algae cells. It was observed that the fluorescence spectrum of the IOM & EOM of algae contained peak A, λ_ex_/λ_em_ = 275/320 nm; peak B, λ_ex_/λ_em_ = 225/325 nm; peak C, λ_ex_/λ_em_ = 275/450 nm; and, peak D, λ_ex_/λ_em_ = 450/350 nm.

A reduction in the intensities of peaks A, C, and D (represent aromatic proteins, fulvic acid substance, and humic acid substance, respectively) observed in the experimental group in Figure 8 indicates that the IOM of the algal cells was changed by Cu_2_O/SiO_2_. The fluorescence intensity of the peak A (protein-like substance) of the EOM of the experimental group algae cells gradually weakened, and the fluorescence intensity of the peak C (fulvic acid substance) and the peak D (humic acid substance) increased. This indicates that *M. aeruginosa* released a large amount of fulvic acid substance and humic acid substance after exposure to Cu_2_O/SiO_2_, and the proteinoid substance in the algae liquid was converted into fulvic acid substance and humic acid substance.

#### 3.3.7. Total Protein

**Figure 9:**
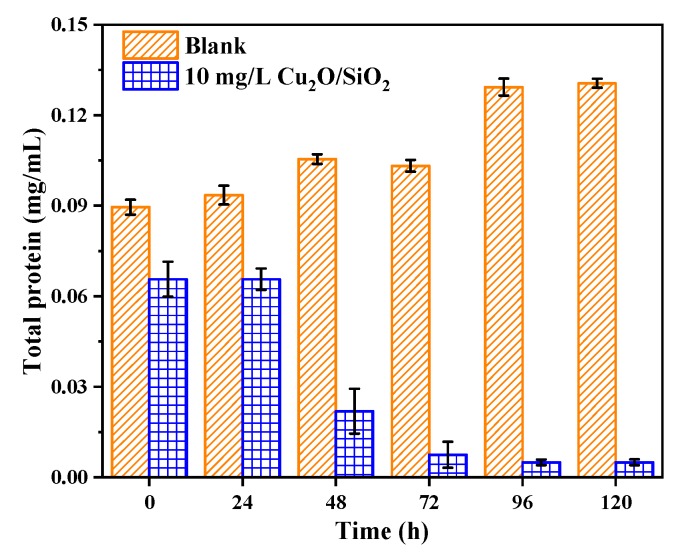
Changes in total protein content of algae during the experiment.

Figure 9 shows the total protein content of *M. aeruginosa* during the experiment. The total protein content of the control group algae increased continuously during the experiment, which was consistent with the trend of OD_680_ and chlorophyll *a* in Section 3.2 and Section 2.2.2. The total protein of the algae cells in the experimental group continued to decrease during the experiment and finally stabilized at 0.00497 mg/mL, which was 5.7% of the initial total protein content. It is speculated that the membrane protein of *M. aeruginosa* was damaged, and the ROS entering the cell destroyed the protein synthesis chain, resulting in a decrease in protein content.

#### 3.3.8. Phycobiliprotein

**Figure 10:**
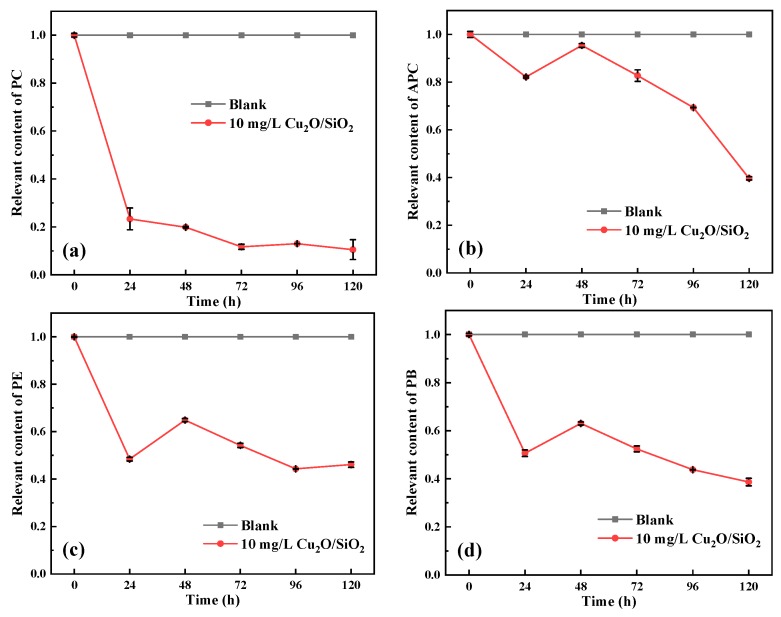
Changes of (**a**) phycocyanin, (**b**) allophycocyanin, (**c**) phycoerythrin, and (**d**) phycobiliprotein content of algae during the experiment.

Figure 10 shows the changes in PB and PBPs during the experiment. It can be seen from Figure 10 that the PC content of *M. aeruginosa* exposed to 10 mg/L showed a downward trend during the experiment, which was 10.5% of the control group at 120 h. A similar trend was observed in the changes of APC, PE, and PB in the algae cells of the experimental group, which were 39.7%, 46.1%, and 38.7% of the control group at 120 h, respectively. Similarly, it is speculated that the ROS produced by Cu_2_O/SiO_2_ might enter the interior of the cell, thereby destroying the phycobiliprotein, thus affecting the photo-harvesting ability of algal cells. This might be one of the mechanisms by which Cu_2_O/SiO_2_ inhibits the growth of *M. aeruginosa*.

#### 3.3.9. Mechanism of Cu_2_O/SiO_2_ Inhibit the Growth of Algal Cells

**Figure 11:**
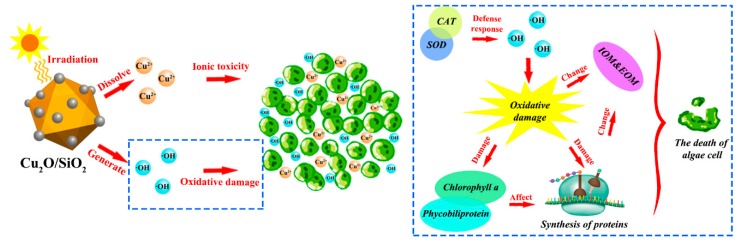
Mechanism of Cu_2_O/SiO_2_ inhibiting algae growth.

In summary, Figure 11 systematically summarizes the mechanism by which Cu_2_O/SiO_2_ inhibits the growth of *M. aeruginosa*. First, Cu_2_O/SiO_2_ will release ·OH under the irradiation of visible light, affecting the activity of SOD and CAT in algae cells, and causing oxidative damage. Second, Cu_2_O/SiO_2_ will damage chlorophyll *a* and phycobiliprotein, which affects the photo-harvesting ability of algae cells. The damage of the photosynthetic system will affect algae cells’ ability to synthesize proteins, resulting in a decrease in total protein content and changes in IOM, and finally cause the death of algae cells. Moreover, Cu_2_O/SiO_2_ will release Cu^2+^ in solution and produce ionic toxicity to *M. aeruginosa*.

## 4. Conclusions

According to the analysis above, it can be concluded that the Cu_2_O/SiO_2_ possesses the characteristics of novel material, low cost, low doses, high inhibitory effects, and good visible light responding. Low doses of Cu_2_O/SiO_2_(10 mg/L) will inhibit the growth of *M. aeruginosa* under low-intensity visible light. In terms of the inhibition mechanism of algae by Cu_2_O/SiO_2_, the Cu^2+^ released from Cu_2_O/SiO_2_ can cause metal toxicity to *M. aeruginosa*. At the same time, Cu_2_O/SiO_2_ will release ·OH in the solution, which will reduce the activity of SOD and increase the activity of CAT, eventually leading to oxidative damage. Besides, Cu_2_O/SiO_2_ will affect the physiological characteristics of algae cells, which causes a decrease in the content of chlorophyll *a*, total protein, and phycobiliprotein, which results in damage, even the death of algae cells. In summary, this study will provide a new direction for the use of Cu_2_O/SiO_2_ to treat harmful cyanobacteria blooms.
